# Biomarkers in newborns with hypoxic-ischemic encephalopathy treated with therapeutic hypothermia

**DOI:** 10.1007/s00381-020-04645-z

**Published:** 2020-05-04

**Authors:** Barbara Michniewicz, Dawid Szpecht, Anna Sowińska, Rafał Sibiak, Marta Szymankiewicz, Janusz Gadzinowski

**Affiliations:** 1 Department of Neonatology, Poznan University of Medical Sciences, Poznan, Poland; 2 Department of Computer Science and Statistics, Poznan University of Medical Sciences, Poznan, Poland; 3 Poznan University of Medical Sciences, Poznan, Poland

**Keywords:** Perinatal asphyxia, Hepatic enzymes, Kidney failure, Troponin T

## Abstract

**Purpose:**

The aim of the presented study was to evaluate the differences between selected biochemical markers in infants with moderate or severe hypoxic-ischemic encephalopathy (HIE) and their impact on patient prognosis.

**Methods:**

A total of 57 cooled newborns were divided into groups according to Sarnat staging of HIE (A, moderate vs. B, severe). The differences between groups were evaluated depending on the mode of delivery, pregnancy and labor complications, gestational age at birth, birth weight, and Apgar score at 1.3 and 5 min. The differences in biochemical biomarkers of HIE (pH, base excess, serum lactate) as well as biomarkers of hepatic injury (aspartate transaminase, (AST), alanine transaminase (ALT), prothrombin time (PT), and activated partial thromboplastin time (APTT)), kidney failure (creatinine, urea), myocardial injury (troponin T (TnT)), levels of fibrinogen, and platelet counts were also examined. Univariate Kaplan-Meier method was used for survival analyses.

**Results:**

The biomarker levels in severe HIE newborns compared with moderate were as follows: pH (7.10 vs. 6.99), serum lactate (22.50 vs. 17.00 mg/dL), AST (109.50 vs. 270.55 IU/L), ALT (27.30 vs. 108.05 IU/L), PT (17.00 vs. 44.20 s), APTT (47.75 vs. 47.90 s), TnT (0.22 vs. 0.85 ng/mL), creatinine (0.68 vs. 1.15 mg/dL), urea (44.55 vs. 73.30 mg/dL), and fibrinogen (1.65 vs. 1.90 mg/dL). Survival analyses showed significantly reduced survival for severe HIE infants (75%) vs. moderate HIE (100%).

**Conclusion:**

In conclusion, the severity of HIE can be evaluated based on selected markers; however, their levels do not correspond with future prognosis of newborns.

## Introduction

Neonatal encephalopathy (NE) is a term used to describe a neurological syndrome caused by a disorder of the brain. Clinical manifestations of NE include features of altered level of consciousness, respiratory depression, seizures, and abnormal muscle tone and reflexes [1]. NE occurs most often as a result of acute hypoxic-ischemic insult, intracranial hemorrhages, numerous metabolic disorders, intrauterine infections, and epileptic syndromes [1, 2]. Hypoxic-ischemic encephalopathy (HIE) is classified as a subtype of NE with characteristic lesions observed in an MRI [1]. The incidence of HIE is estimated to be between 1.3 and 1.7 per 1000 live births [3].

HIE is associated with increased risk of neonatal mortality; 15–20% of neonates die due to complications of perinatal asphyxia [3]. Moreover, HIE often leads to sustained severe and irreversible brain damage. Further observations of infants affected by perinatal asphyxia revealed that 27–33% of them at an age of 6 to 7 years will develop features of intellectual disability (IQ under 70 and deficits in adaptive behaviors) [4]. Patients frequently present a broad spectrum of other neurological symptoms, such as motor dysfunction, hearing loss, impaired vision, epilepsy, and behavioral problems, which occur as a consequence of a perinatal hypoxic-ischemic episode. A diagnosis of cerebral palsy was confirmed in 17–29% of children with a history of perinatal asphyxia [4–6].

Evaluation of the severity of HIE is a crucial determinant in predicting the future outcomes for children with HIE. Physical examination combined with neurological assessment, performed according to Sarnat staging [7], gives us reliable information about the future prognosis. Previous studies revealed a group of plasma biomarkers (S100B, UCH-L1, total Tau, neuron-specific enolase, brain-derived neurotrophic factor (BDNF), and inflammatory cytokines), which can be useful in assessing the extent of brain damage. Furthermore, baseline Tau and BDNF levels correlate with worse 1-year outcomes [8, 9]. However, perinatal asphyxia can disrupt functioning of other internal organs. Measurement of the concentration of specific serum and urinary biomarkers can be helpful in early detection of hypoxic-ischemic injury of the kidneys, liver, and heart [10–12].

Meta-analyses of previous studies showed that therapeutic hypothermia (TH) reduces mortality and neurodevelopmental disability in groups of term or late preterm newborns with suspicion of peripartum asphyxia [13]. Before initiation of therapeutic cooling, infants must fulfill certain eligibility criteria. Cooling should be performed according to standardized selective head cooling (SHC) or whole-body cooling (WBC) protocols. Results of recent clinical trials revealed that both methods—SHC and WBC—have proven to have a neuroprotective effect [14–16]. As a result, hypothermia has emerged as the standard of care to infants with moderate–severe HIE.

Taking into consideration that early evaluation of severity of HIE, as well as early detection of potential multiorgan injury caused by asphyxia in diagnosed neonates, can be helpful in the prognosis of newborns’ outcomes; the aim of the presented study was to assess whether the differences between selected biochemical markers in infants with moderate or severe HIE affect the future patient’s prognosis.

## Methods

### Patients and inclusion criteria

A retrospective study of 57 neonatal patients with diagnosed HIE was conducted in the Department of Neonatology at Poznan University of Medical Sciences between 2009 and 2017. Therapeutic hypothermia was introduced into the standard clinical practice for infants born ≥ 36 weeks of gestation with suspected perinatal asphyxia within 6 h after birth. Before the application of TH, newborns with presumed HIE were evaluated in accordance with specific eligibility criteria. All patients enrolled in the study met both treatment criteria A and B. Criterion A included at least one of the following conditions: (1) acidosis of arterial blood or the blood obtained from the umbilical cord (pH < 7.00, within 60 min after birth) or base excess (BE) ≥ 16 mmol/l; (2) Apgar score ≤ 5 at 10 min after birth; and (3) resuscitation ≥ 10 min after birth. Criterion B included patients with moderate-to-severe HIE according to Sarnat staging, especially with a state of impaired consciousness (lethargy or coma), and with at least one additional feature such as decreased muscle tone, abnormal stimulus response (oculomotor nerve impairment, abnormal pupillary light reflex, weak or absent suck reflex), and clinically proven seizures. Before induction of the cooling procedure, the patient’s brain activity was assessed for at least 20 min using an amplitude-integrated electroencephalogram (aEEG). All of the aEEG records presented features of moderate or severe brain dysfunction and seizures. Moderately abnormal aEEGs were defined as visible changes in the activity band (discontinuous background pattern—lower margin of the band < 5 μV). The presence of burst suppression, low continuous voltage, or flat tracing in recorded activity band was classified as patterns of severely abnormal aEEG [17, 18].

### Cooling protocol

Patients were cooled with a selective head cooling Cool-Cap system (14 neonates) and a whole-body cooling device (Nz Techno, Tecotherm, 43 neonates). After application of therapeutic hypothermia, neonates were cooled for 72 h. Selective head cooling (SHC) and whole-body cooling (WBC) require a continuous body (rectal) temperature measurement. During SHC, the core body temperature was reduced to 34.5 °C. In a group of patients cooled with WBC, core temperature was maintained at 33.0–33.5 °C. After that, the patients were slowly rewarmed (0.5 °C/h) to 36.5 °C.

### Analyzed parameters

Newborns were divided into groups according to Sarnat staging of HIE (group A, grade II moderate vs. group B, grade III severe). The differences between groups were evaluated depending on the mode of delivery, pregnancy and labor complications, gestational age at birth, birth weight, and Apgar score at 1.3 and 5 min. The differences in biochemical plasma biomarkers of HIE were also evaluated. The comparison of metabolic acidosis parameters (pH and base excess) was performed on fetal umbilical cord arterial blood samples obtained immediately after birth. The measurements of pH, base excess, and level of serum lactate were carried out on two additional specimens of blood obtained from umbilical vessels after 1 h and at latest after 4 h after birth. The levels of plasma biomarkers of hepatic injury (aspartate transaminase (AST), alanine transaminase (ALT), prothrombin time (PT), and activated partial thromboplastin time (APTT)), kidney failure (creatinine, urea), myocardial injury (troponin T, TnT), levels of fibrinogen, and platelet counts were also examined. All blood samples were collected in the first 24 h after birth.

### Statistical analysis

Statistical analysis was performed using Statistica. Descriptive statistics are presented as percentages for categorical variables, mean and standard deviation for continuous, normally distributed variables, and median with range for continuous variables with asymmetry of distribution. Normality of the distributions was checked by Shapiro-Wilk test. The prevalence of variables was assessed by the chi square test, the Fisher exact test, or Fisher-Freeman test. Continuous variables were compared using the Mann-Whitney *U* test, Student’s *t* test, or Cochran-Cox test.

## Results

A total of 57 neonatal patients were enrolled in the study. Table 1 shows the baseline characteristics of the 57 neonates analyzed (Table 1). The vast majority of them (45 out of 57) were transferred from other institutions to the tertiary referral hospital within 6 h after birth. Pregnancy or labor complications occurred in 45 out of 57 (78.9%) of the patients. The other most common complications were placental abruption (15 out of 57 cases), meconium-stained amniotic fluid (12 out of 57), intrauterine infection (8 out of 57), and gestational diabetes (3 out of 57). Two cases of pregnancy-induced hypertension, umbilical cord compression, and problems with fetal expulsion were also noted.

**Table 1:** Demographic and clinical characteristics of enrolled infants

	Group with moderate HIE *n* = 27 (%)	Group with severe HIE *n* = 30 (%)	*p* value
Gender			0.678^a^
Male	13	18	
Female	14	12	
Gestational age (week)	36 (36–42)	38 (36–41)	0.061^d^
Birth weight (gram)	3314.25 ± 507.78	3306.16 ± 641.89	0.958^e^
Mode of delivery			0.397^b^
Forceps	2	0	
Vaginal	3	7	
Vacuum	1	1	
Cesarean section	21	22	
Inborn	6	6	0.837^a^
Outborn	21	24	
Deaths			0.0049^c^
Yes	0	8	
No	27	22	

^a^Chi-squared test

^b^Fisher Freeman Halton

^c^Fisher’s exact test

^d^Mann-Whitney

^e^Student’s *t* test

The neurological examination provided information about clinical staging of HIE. Patients were divided into groups according to Sarnat staging: 27 out of 57 (47.4%) met the criteria of a group HIE - A moderate; the rest of them 30 out of 57 (52.6%) were classified as cases of severe HIE - B. Statistically significant differences between the groups of moderate and severe HIE were noted (Table 2). We noticed the level of serum lactate from the first after 1 h (A, 22.50 mg/dL (range 6.80–162.00) vs. B, 17.00 mg/dL (range 0.00–180.00)) and the second after at latest 4 h after birth specimen (A, 53.50 mg/dL (range 2.00–164.00) vs. B, 115 mg/dL (range 14.50–186.00), *p* = 0.027) and results of base excess in material obtained from two samples (A, − 15.02 ± 5.86 vs. B, − 19.03 ± 5.53, *p* = 0.016 and A, − 9.06 ± 5.80 vs. B, − 14.34 ± 5.62, *p* = 0.001). Statistically significant results were also observed in the levels of hepatic injury biomarkers—AST (A, 109.50 IU/L (range 21.10–819.20) vs. B, 270.55 IU/L (range 66.10–3175.00), *p* = 0.0004), ALT (A, 27.30 IU/L (range 8.00–571.00) vs. B, 108.05 IU/L (range 16.60–1195.30), *p* = 0.0008), PT (A, 17.0 s (range 10.00–44.20) vs. B, 24.40 s (range 14.50–320.00), *p* = 0.0004), APTT (A, 47.75 s (range 34.40–92.70) vs. B: 47.90 s (range 30.30–400.00), *p* = 0.302); myocardial injury biomarker—TnT (A, 0.22 ng/mL (range 0.06–1.62) vs. B, 0.85 ng/mL (range 0.05–10.00), *p* < 0.0001); and kidney failure markers—creatinine (A, 0.68 mg/dL (range 0.23–2.26) vs. B, 1.15 mg/dL (range 0.63–3.16), *p* < 0.0001) and urea (A, 44.55 mg/dL (range 19.30–108.70) vs. B, 73.30 mg/dL (range 32.20–137.90), *p* = 0.002). Also, different levels of fibrinogen were noted (A, 1.65 mg/dL (range 0.79–3.61) vs. B, 1.90 mg/dL (range 0.30–6.90), *p* = 0.274) and thrombocytopenia (platelet count < 100,000/mm^3^) was observed in 8/27 cases of moderate HIE and in 20/29 cases of severe HIE (*p* = 0.003).

**Table 2:** The levels of selected biomarkers in moderate or severe HIE newborns

	HIE		
Biomarkers	Moderate	Severe	*p* value
pH from the umbilical cord 1	6.89 (6.65–7.28)	6.81 (6.59–7.29)	0.0627^c^
Base excess 1	− 17.24 ± 7.50 mmol/L	− 20.67 ± 7.67 mmol/L	0.173^a^
pH from the umbilical cord 2	6.86 (6.68–7.28)	6.89 (6.61–7.42)	0.693^c^
Base excess 2	− 15.84 ± 5.40 mmol/L	− 18.38 ± 5.47 mmol/L	0.305^a^
pH from the blood 1	7.10 ± 0.18	6.99 ± 0.22	0.056^a^
Base excess 1	− 15.02 ± 5.86 mmol/L	− 19.03 ± 5.53 mmol/L	0.016^b^
Serum lactate 1	22.50 (6.80–162.00) mg/dL	17.00 (0.00–180.00) mg/dL	0.979^c^
pH from the blood 2	7.25 (7.04–7.43)	7.21 (6.59–7.46)	0.446^c^
Base excess 2	− 9.06 ± 5.80 mmol/L	− 14.34 ± 5.62 mmol/L	0.001^b^
Serum lactate 2	53.50 (2.00–164.00) mg/dL	115.00 (14.50–186.00) mg/dL	0.027^c^
TnT	0.22 (0.06–1.62) ng/mL	0.85 (0.05–10.00) ng/mL	0.000004^c^
AST	109.50 (21.10–819.20) IU/L	270.55 (66.10–3175.00) IU/L	0.0004^c^
ALT	27.30 (8.00–571.00) IU/L	108.05 (16.60–1195.30) IU/L	0.0008^c^
Creatinine	0.68 (0.23–2.26) mg/dL	1.15 (0.63–3.16) mg/dL	0.00006^c^
Urea	44.55 (19.30–108.70) mg/dL	73.30 (32.20–137.90) mg/dL	0.002^c^
INR	1.44 (0.97–3.96)	2.04 (1.07–7.54)	0.004^c^
APTT	47.75 (34.40–92.70) s	47.90 (30.30–400.00) s	0.302^c^
PT	17.00 (10.00–44.20) s	24.40 (14.50–320.00) s	0.0004^c^
Fibrinogen	1.65 (0.79–3.61) mg/dL	1.90 (0.30–6.90) mg/dL	0.274^c^
Apgar 1	1.0 (0.0–6.0)	0.5 (0.0–5.0)	0.290^c^
Apgar 3	3.0 (0.0–6.0)	2.0 (0.0–6.0)	0.116^c^
Apgar 5	4 (0.0–8.0)	3 (0.0–8.0)	0.081^c^

*ALT* alanine transaminase, *APTT* activated partial thromboplastin time, *AST* aspartate aminotransferase, *INR* international normalized ratio, *PT* prothrombin time, *TnT* troponin T

^a^Student’s *t* test

^b^Cochran-Cox

^c^Mann-Whitney

### Survival analyses

**Fig. 1:**
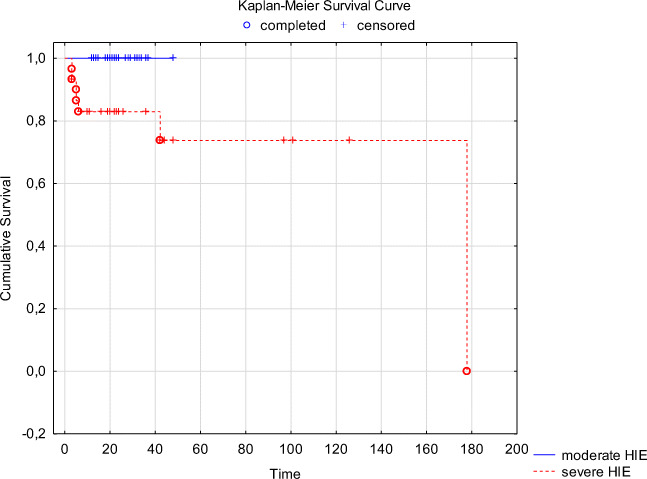
Survival analyses

Univariate Kaplan-Meier’s analysis showed significantly reduced survival for cases with severe HIE (group B) compared with that of patients with moderate HIE (group A). While the survival probability for newborns with moderate HIE is 100%, for children with severe HIE, it is only 75% (Fig. 1). However, multivariate analyses of all 57 neonates revealed that biochemical markers of hypoxia such as lactate (*p* = 0.100826), TnT (*p* = 0.218603), AST (*p* = 0.957588), ALT (*p* = 0.285924), creatinine (*p* = 0.794386), or urea (*p* = 0.306928) had no effect on the probability of survival of patients with HIE (Table 3).

**Table 3:** Multivariate analyses of biochemical markers of hypoxia in HIE newborns in terms of survival

Dependent variable: discharge day Variable cutting rate, trimmed 0-not, 1-yes *p* = 0.05878	*p* value	HR	Lower 95% CI	Upper 95% CI
Lactate 2 1- ≤ 22 2-22-145 3- > 145	0.100826	0.236930	0.042427	1.3231
TnT 1- ≤ 0.20 2-0.20-0.94 3- > 0.94	0.218603	2.615777	0.565349	12.1028
AST 1- ≤ 93.4 2-93.4-552.4 3- > 552.4	0.957588	0.914120	0.033404	25.0157
ALT 1- ≤ 25.3 2-25.3-238.7 3- > 238.7	0.285924	5.132402	0.254502	103.5024
Creatinine 1- ≤ 0.64 2-0.64-1.57 3- > 1.57	0.794386	0.820758	0.185817	3.6253
Urea 1- ≤ 39.3 2-39.3-84.6 3- > 84.6	0.306928	0.434016	0.087523	2.1522

## Discussion

Perinatal asphyxia, leading to moderate or severe HIE, causes multiorgan injury and dysfunction not only of the brain, but also the heart, liver, kidneys, and bone marrow [19, 20]. These pathophysiological disorders are related to the severity of encephalopathy and subsequent neurodevelopmental outcomes [21, 22]. Therefore, evaluation of severity of HIE, as well as early detection of each potential injury in diagnosed neonates, may help predict morbidity and aid clinical management.

Different kinds of markers have been studied to identify perinatal hypoxia and neural injury [9, 23]; herein, we demonstrated differences between levels of selected biomarkers of hepatic, kidney, and myocardial injury in newborns with moderate or severe HIE treated with TH. The levels of all tested biomarkers tend to stray further from their normal range in the severe HIE group. Each of these biomarkers is discussed in the following sections.

### Measurements of pH, base excess, and serum lactate

In the presented study, besides neurological examination, serum lactate was used to predict the severity and outcome of neonatal HIE. Interestingly, we noted no significant differences between tested groups in first specimen taken in the first hour of life (*p* = 0.997), however higher levels of serum lactate in the severe HIE infants compared with the moderate in second specimen taken at latest after 4 h after birth (*p* = 0.027). This is in accordance with studies of Helijic et al. [24] as well as Murray et al. [25], who suggest that first lactate value taken after birth do not predict severity of HIE. It has been demonstrated that a plasma lactate concentration taken during first 24 h of life was associated with moderate or severe HIE [26], and a significantly higher level of lactate was observed in infants with poor outcome compared with those with favorable outcomes [27]. We observed also significant reduction of umbilical cord pH in both tested groups as well as base deficit, which can also be used as a prognostic factor for unfavorable short-term outcomes in newborns, according to Ahmadpour-Kacho et al. [28].

### Biomarkers of hepatic injury

Ischemic hepatic injury occurs in about 40–60% of the diagnosed infants with HIE [8]. ALT and AST levels increase as a result of hypoxia organ damage and are sensitive markers of impaired liver membrane [29]. In our study, we showed that the levels of both biomarkers reflecting hepatocellular integrity, AST as well as ALT, differed according to HIE grade. Infants with severe HIE had significantly higher hepatic enzyme concentrations compared with that of moderate HIE infants (*p* = 0.0004). Moreover, greater differences between moderate and severe HIE babies (two times more vs. four times more) were observed in the case of ALT, which is considered to be a more specific marker for hepatic injury than AST [30]. The results of our observations are consistent with several previously published data [10, 31, 32], where authors noted not only an increase of ALT and AST concentrations but also significant correlation between their levels with the severity of HIE in newborns. Additionally, we tested PT, which is the biomarker reflecting hepatic synthetic function, and we noted longer prothrombin time in the group of infants with severe HIE (*p* = 0.0004), which confirmed the observations obtained in another cohort study [31]. Interestingly, only a few papers tested the hepatoprotective effect of the TH in perinatal HIE babies. A meta-analysis of six randomized controlled trials, which included 975 infants, showed no significant hepatoprotective effect of therapeutic hypothermia (relative risk 0.88 (95% CI 0.74 to 1.05)) [13]. In turn, Muniraman et al. observed inconsistent results for individual hepatic markers, with significantly lower peak ALT concentrations in the hypothermia group, but no difference for AST concentrations and prothrombin time [10].

### Biomarkers of kidney failure

If kidney function is impaired, urea and creatinine cannot be removed from the body, and their blood levels increase. Frequently impaired renal function occurs as a result of acute kidney injury (AKI), which concerns 7–72% of asphyxiated infants and alters their urinary biomarker profile [11]. It is well-known that serum urea and creatinine levels are significantly elevated in the HIE patient group in comparison with the control group [33, 34]. In our study, there was a significant increase regarding urea and creatinine levels in the severe HIE group when compared with that of the moderate HIE group of infants (*p* = 0.002 and *p* < 0.001, respectively). These findings mean that kidney biomarkers, such as urea or creatinine concentrations, correlated with the degree of HIE. Similarly, a rising trend in the concentrations of blood urea and creatinine as HIE staging of neonates progressed was also observed by El-Gamasy et al., Gopal et al., and Alaro et al. [35–37]. Interestingly, studies of Park et al., confirmed by El-Gamasy et al. in 2018, revealed an association between severity of HIE and the development of AKI in asphyxiated neonates [38]. Generally, the greater the degree of HIE, the higher the incidence of acute renal failure (AKI). Kaur et al. showed that AKI developed in one out of 11 infants (9.1%) with moderate asphyxia and in 12 of 25 (48%) with severe asphyxia [39]. Moreover, Alaro et al. noted a 15-fold increased risk of developing AKI in HIE III compared with HIE I [37]. To our knowledge, there is no specific data focused on the protective action of TH on the kidneys.

### Biomarkers of myocardial injury (TnT)

Myocardial damage occurs in 28 to 73% of neonatal asphyxia cases and is the main cause of neonatal mortality associated with hypoxia–ischemia [40]. Asphyxiated neonates have higher cardiac TnT concentrations than that of controls [39]. Several studies have confirmed that serum level of TnT is a specific biomarker of myocardial injury caused by neonatal asphyxia at an early stage [41]. In the presented study, we observed that severe HIE infants had approximately four-fold higher concentration of TnT compared with that of the group with moderate HIE (*p* < 0.0001). Also, Gunes et al. found that TnT concentrations in the first hours of life were significantly higher in HIE grade 3 compared with that in grades 1 and 2 [42]. Significant relation with increasing cTnT values and increasing grades of HIE were also observed by Joseph et al. [43]. Another study investigated levels of cardiac troponin I (cTnI) in HIE. Türker et al. demonstrated that newborns with moderate–severe asphyxia had significantly higher cTnI levels in cord blood and in venous blood compared with those with no or only mild asphyxia [44]. Moreover, Shastri et al. concluded that in asphyxiated neonates, cTnI concentrations within 36 h of birth correlated strongly with clinical grade of HIE [12]. Importantly, in a retrospective study by Liu et al., the level of cardiac troponin was decreased following hypothermia. The authors suggest that cTnI levels lower than 0.15 ng/mL for cooled infants at 24 h age predict improved outcomes [45].

### Levels of fibrinogen and platelets

Thrombocytopenia, defined as a platelet count < 150,000/mL, is associated with increased risk of bleeding, and severe thrombocytopenia (platelet count < 50,000/mL) is associated with significant morbidity [46]. As a result, it is important to identify at-risk infants, and if needed, to initiate therapy to prevent complications. In most cases, thrombocytopenia develops in neonates with HIE as a result of hypothermia therapy [47]. In our results, we observed thrombocytopenia in 8/27 cases of moderate HIE and in 20/29 cases of severe HIE (*p* = 0.003), which suggests a correlation between morbidity and severity of HIE. These findings were in agreement with data from Bala et al., who showed reduced platelet counts in HIE and noted that during the first 48 h of life it is related to severity of HIE [48].

As the endpoint, we tried to assess whether severity of HIE and selected biomarkers can be helpful in the prognosis of infants’ outcomes. Although the results of our analysis showed significantly reduced survival for cases with severe HIE compared with that of patients with moderate HIE, there was no correlation between biomarkers and mortality. There are data showing that several biochemical markers, including serum S100b and neuron-specific enolase (NSE), are predictors of death, neurological abnormalities, or MRI-detected brain injury in hypothermia-treated HIE [49], but their levels depend on the timing of sampling, and their prognostic value is uncertain [20]. The limitation of our study may be due to the relatively small number of infants studied, making them underpowered for assessing correlations between given biomarkers and infants’ survival.

In conclusion, the severity of the hypoxic-ischemic injury can be evaluated based on selected markers; however, their levels do not correspond with future prognosis of newborns.
